# The role of serum zinc and MicroRNAs in osteoporosis: insights into bone metabolism and potential diagnostic markers

**DOI:** 10.1186/s12891-025-08970-9

**Published:** 2025-08-09

**Authors:** Parastoo Karimi, Mohammad Reza Mirzaei, Farzaneh Arami, Zahra Bagheri-Hosseinabadi, Mitra Abbasifard, Saeed Mohammadi-Hosseinabad, Zahra Jalali, Mehdi Mahmoodi, Mohammad Reza Hajizadeh

**Affiliations:** 1 Molecular Medicine Research Center, Research Institute of Basic Medical Sciences, Rafsanjan University of Medical Sciences, Rafsanjan, Iran; 2 Department of Clinical Biochemistry, School of Medicine, Rafsanjan University of Medical Sciences, Rafsanjan, Iran; 3 Immunology of Infectious Diseases Research Center, Research Institute of Basic Medical Sciences, Rafsanjan University of Medical Sciences, Rafsanjan, Iran; 4 Department of Internal Medicine, Ali-Ibn Abi-Talib Hospital, School of Medicine, Rafsanjan University of Medical Sciences, Rafsanjan, Iran; 5 Department of Clinical Biochemistry, Afzalipour School of Medicine, Kerman University of Medical Sciences, Kerman, Iran

**Keywords:** Zinc, MicroRNA, Bone metabolism, Osteoporosis

## Abstract

**Background:**

Zinc (Zn) levels are reportedly lower in osteoporosis patients and may be linked to bone health. MicroRNAs (miRNAs) have also been implicated in the regulation of bone remodeling. This study aims to investigate the role of selected miRNAs and their potential interplay with Zn in the pathogenesis of osteoporosis.

**Methods:**

In this case-control study, 50 patients with osteoporosis and 50 healthy controls were recruited. The serum transcript levels of miRNAs were assessed using Real-time PCR.

**Results:**

Levels of Zn was significantly (*P* = 0.0047) lower in the serum samples from osteoporosis patients in comparison to the healthy controls. The transcript level of miR-34a-5p (Fold change = 2.59, *P* = 0.008) and miR-335-5p (Fold change = 1.90, *P* = 0.013) was upregulated significantly in the serum samples of patients with osteoporosis compared to that of the healthy controls. Zn level in osteoporosis patients had significant negative correlation with transcript levels of miR-335-5p (*r*= -0.24, *P* = 0.037). Zn level had positive correlation with Z and T-scores and BMD of hip, spine, and femur. A negative correlation (*r*= -0.28 *P* = 0.044) was detected between transcript level of miR-34a-5p and femur BMD. There was a significant negative correlation between transcript level of miR-335-5p and hip BMD (*r*= -0.19 *P* = 0.038). miR-34a-5p and miR-335-5p showed diagnostic potential.

**Conclusions:**

The findings suggest a potential interplay between Zn and miRNAs in the regulation of bone metabolism in osteoporosis patients. Zn and miRNAs may serve as biomarkers for bone health and a therapeutic target in osteoporosis management.

**Supplementary Information:**

The online version contains supplementary material available at 10.1186/s12891-025-08970-9.

## Introduction

Osteoporosis is a chronic metabolic bone disorder characterized by reduced bone mineral density (BMD) and microarchitectural deterioration, leading to increased bone fragility and susceptibility to fractures [1]. Globally, osteoporosis affects millions of individuals, particularly postmenopausal women and the elderly, imposing a significant healthcare burden [2]. The etiology of osteoporosis is multifactorial, encompassing genetic predisposition, hormonal changes, nutritional deficiencies, and lifestyle factors. Despite advances in diagnostic tools and treatment strategies, a deeper understanding of the molecular mechanisms underlying osteoporosis remains critical for developing more effective interventions [3].

MicroRNAs (miRNAs), a class of small non-coding RNAs, are pivotal regulators of post-transcriptional gene expression. They influence numerous biological processes, including cell differentiation, proliferation, and apoptosis [4]. Emerging evidence suggests that miRNAs play a significant role in bone metabolism by modulating pathways involved in osteoblast and osteoclast activity [5]. Among these, miR-34a-5p, miR-335-5p, and miR-150-5p have garnered attention due to their involvement in key signaling pathways affecting bone homeostasis. Dysregulation of these miRNAs has been implicated in osteoporosis pathophysiology, suggesting their potential as biomarkers for diagnosis and disease progression monitoring [6]. As an instance, studies indicated involvement of miR-150-5p in osteogenic differentiation of bone marrow-derived mesenchymal stem cells (BM-MSC) through regulation of the p38/MAPK signaling pathway [7].

Zinc (Zn) is an essential trace element integral to various physiological processes, including enzymatic functions, immune regulation, and bone metabolism [8]. Zn is critical for osteoblast proliferation and collagen synthesis, both of which are essential for bone matrix formation [9, 10]. Reduced Zn levels have been observed in individuals with osteoporosis, highlighting its potential role in disease development [11]. Despite its recognized importance, the relationship between Zn and miRNA expression in osteoporosis remains inadequately explored. Understanding the interplay between Zn and miRNAs could provide novel insights into the molecular underpinnings of osteoporosis.

In this study, we aim to assess the serum levels of Zn and specific miRNAs (including miR-34a-5p, miR-335-5p, and miR-150-5p) in patients with osteoporosis and healthy controls. By investigating the potential correlation between these biomarkers, we seek to elucidate their combined role in the pathogenesis of osteoporosis. This research may pave the way for identifying novel diagnostic tools and therapeutic targets, contributing to more personalized and effective management strategies for osteoporosis.

### Materials and methods

#### Study participants

This study was conducted on participants referred to the outpatient rheumatology clinic at Ali-Ibn Abi-Talib Hospital, Rafsanjan University of Medical Sciences, Rafsanjan, Iran. Participants were selected based on specific inclusion criteria, excluding individuals with conditions such as liver or kidney failure, thyroid or parathyroid disorders, hematological conditions (e.g., anemia, thrombocytopenia, leukopenia), malignancies, autoimmune diseases (e.g., ankylosing spondylitis, rheumatoid arthritis, systemic lupus erythematosus), hip or spine fractures, active infections, or a history of blood transfusion within the past year. Additionally, individuals taking medications such as bisphosphonates, selective estrogen receptor modulators, or steroids were excluded. However, the use of supplements like vitamin D and calcium was permitted.

In this study, BMD measurements were obtained for all participants at the hip, femoral neck, and L1-L4 lumbar spine while they were positioned supine. The assessments were performed using the Stratos device (DMS Imaging, France) via the dual-energy X-ray absorptiometry (DXA) technique. According to World Health Organization (WHO) criteria, participants with a T-score of < −2.5 were classified as having osteoporosis, while those with a T-score > −1 were categorized as controls [12]. Based on these criteria, 50 cases with osteoporosis and 50 healthy controls were included in the study. Following participant selection, 5 mL of whole blood was collected from each individual for serum isolation and further analysis. Serum was separated immediately after collection by centrifugation and stored at −80 °C for subsequent analysis. Baseline demographic and biochemical characteristics of the participants are summarized in Table 1. This study was approved by the Ethics Committee of Rafsanjan University of Medical Sciences (Approval Code: IR.RUMS.REC.1403.030), and written informed consent was obtained from all participants prior to their inclusion in the study.

**Table 1:** Baseline data, demographics and laboratory measurements of the study participants

Variable	Osteoporosis (*n* = 50)	Control (*n* = 50)	OR (95% CI)	*P* value
Sex (Male/Female); n (%)	9 (18%)/41 (82%)	9 (18%)/41 (82%)	1.00 (0.36 to 2.77)	0.999
Smoking (Yes/No); n (%)	6 (12%)/44 (88%)	7 (14%)/43 (86%)	0.83 (0.26 to 2.69)	0.766
Residency (Urban/Rural); n (%)	45 (90%)/5 (10%)	44 (88%)/6 (12%)	1.22 (0.34 to 4.31)	0.749
Hypertension (Yes/No); n (%)	12 (24%)/38 (76%)	10 (20%)/40 (80%)	1.26 (0.48 to 3.26)	0.629
Diabetes mellitus (Yes/No); n (%)	4 (8%)/46 (92%)	2 (4%)/48 (96%)	2.08 (0.36 to 11.94)	0.408
Vitamin D supplementation (Yes/No); n (%)	46 (92%)/4 (8%)	7 (14%)/43 (86%)	70.64 (19.31 to 258.41)	**< 0.0001**
Calcium supplementation (Yes/No); n (%)	46 (92%)/4 (8%)	2 (4%)/48 (96%)	276.00 (48.20 to 1580.22)	**< 0.0001**
Job (working/not working); n (%)	32 (64%)/18 (36%)	35 (70%)/15 (30%)	0.76 (0.33 to 1.75)	0.523
Marital status (married/single); n (%)	40 (80%)/10 (20%)	41 (82%)/9 (18%)	0.87 (0.32 to 2.38)	0.798
Education (educated/non-educated); n (%)	47 (94%)/3 (6%)	48 (96%)/2 (4%)	0.65 (0.10 to 4.08)	0.648
Familial history of Osteoporosis (Yes/No); n (%)	11 (22%)/39 (78%)	-	-	**-**
Bone fracture history (Yes/No); n (%)	9 (18%)/41 (82%)	-	-	**-**
Familial History of bone fracture (Yes/No); n (%)	7 (14%)/43 (86%)	-	-	**-**
Age (Year); mean ± SD	54.2 ± 7.5	53.7 ± 8.6	-	0.358
BMI (kg/m^2^); mean ± SD	25.14 ± 3.10	25.80 ± 5.02	-	0.547
Menarche age (Year); mean ± SD	12.47 ± 1.52	12.08 ± 1.57	-	0.970
Menopause duration (Year); mean ± SD	8.69 ± 2.08	8.64 ± 1.80	-	0.209
WBC (cells/mm^3^); mean ± SD	6128 ± 875	5569 ± 932	-	**0.027**
Platelet count (cells/mm^3^); mean ± SD	189,000 ± 12,500	195,000 ± 10,700	**-**	0.201
Hemoglobin (g/dl); mean ± SD	13.25 ± 2.08	14.46 ± 2.84	**-**	0.352
RBC count (million cells/mm^3^); mean ± SD	4.64 ± 1.53	5.45 ± 1.67	-	0.411
AST (IU/L); mean ± SD	27.12 ± 5.41	22.16 ± 5.76	-	0.014
ALP (IU/L); mean ± SD	139.14 ± 31.30	124.46 ± 24.63	-	0.016
ALT (IU/L); mean ± SD	30.52 ± 8.50	27.39 ± 7.61	-	0.025
LDH (IU/L); mean ± SD	212.48 ± 42.32	184.79 ± 38.97	-	0.001
CRP (mg/L); mean ± SD	3.15 ± 1.12	0.84 ± 0.23	**-**	**0.001**
ESR (mm/h); mean ± SD	15.74 ± 4.16	2.32 ± 1.77	**-**	**0.001**
FBS (mg/dl); mean ± SD	102.11 ± 8.23	98.23 ± 9.03	-	0.114
Cholesterol (mg/dl); mean ± SD	126.31 ± 24.56	125.17 ± 25.83	-	0.680
TG (mg/dl); mean ± SD	118.23 ± 18.64	113.41 ± 21.66	-	0.179
LDL (mg/dl); mean ± SD	105.13 ± 15.79	102.51 ± 12.79	-	0.513
HDL (mg/dl); mean ± SD	46.79 ± 6.32	49.64 ± 7.26	-	0.794
Creatinine (mg/dl); mean ± SD	1.43 ± 0.24	1.21 ± 0.30	-	0.550
BUN (mg/dl); mean ± SD	13.79 ± 4.58	12.64 ± 3.40	-	0.731
Z-score Hip; mean ± SD	−1.9 ± 0.7	0.1 ± 0.0	-	**< 0.0001**
T-score Hip; mean ± SD	−1.7 ± 0.1	0.7 ± 0.1	-	**< 0.0001**
Z-score Spine; mean ± SD	−3.1 ± 0.5	0.1 ± 0.0	-	**< 0.0001**
T-score Spine; mean ± SD	−3.3 ± 0.4	0.5 ± 0.1	-	**< 0.0001**
Z-score Femur; Mean ± SD	−2.8 ± 0.5	0.1 ± 0.0	-	**< 0.0001**
T-score Femur; Mean ± SD	−3.1 ± 0.5	0.2 ± 0.1	-	**< 0.0001**
Spine BMD (g/cm2); Mean ± SD	0.794 ± 0.206	1.514 ± 0.314	-	**< 0.0001**
Hip BMD (g/cm2); Mean ± SD	0.627 ± 0.109	1.406 ± 0.210	-	**< 0.0001**
Femur BMD (g/cm2); Mean ± SD	0.910 ± 0.213	1.631 ± 0.190	-	**< 0.0001**

*BMI,* Body-mass index, *WBC,* White blood cell, *ALP,* Alkaline phosphatase, *AST,* Aspartate aminotransferase, *ALT*, Alanine aminotransferase, *LDH*, Lactate dehydrogenase, *CRP*, C-reactive protein, *ESR*, Erythrocyte sedimentation rate, *FBS*, Fasting blood sugar, *TG*, Triglyceride, *LDL*, Low-density lipoprotein, *HDL*, High-density lipoprotein, *BUN*, Blood urea nitrogen, *BMD,* Bone mineral density, *SD*, Standard deviation, *OR*, Odds ratio, *CI*, Confidence interval

*Bold values show statistically significant comparisons

#### Laboratory measurements

The serum levels of alkaline phosphatase (ALP), aspartate aminotransferase (AST), alanine aminotransferase (ALT), lactate dehydrogenase (LDH), fasting blood sugar (FBS), triglycerides (TG), low-density lipoprotein (LDL), high-density lipoprotein (HDL), creatinine, and blood urea nitrogen (BUN) were analyzed using enzymatic colorimetric methods following overnight fasting. The erythrocyte sedimentation rate (ESR) was assessed through an automated kinetic photometric method, while C-reactive protein (CRP) levels were quantified using the nephelometric technique. Furthermore, the complete blood count (CBC) was performed to determine blood cell counts, utilizing the Sysmex KX-21 N Hematology Analyzer (Sysmex, Japan). The colorimetric method was used to measure the levels of Zn in serum of subjects by biochemistry autoanalyzer (BT3000, Biotecnica, Italy).

#### Determination of miRNA expression levels

Total RNA was extracted from serum samples using Trizol reagent (TaKaRa, South Korea). The RNA’s quality and purity were assessed with a NanoDrop spectrophotometer (ND-2000, Thermo Fisher Scientific, USA). Complementary DNA (cDNA) was synthesized from the extracted RNA using miScript II RT Kit (Qiagen, Germany), following the manufacturer’s protocol.

The relative expression levels of miR-34a-5p, miR-335-5p, and miR-150-5p were determined using a real-time PCR approach with SYBR Green chemistry (Ampliqon, Denmark) and specific primers (detailed in Supplementary Table 1). Each PCR reaction contained 3 µL of cDNA template, 5 µL of master mix, 1 µL of each forward and reverse primer, and 10 µL of RNase-free distilled water, resulting in a final reaction volume of 20 µL. The reactions were performed in a Rotorgene thermal cycler (Rotorgene, Malaysia) with the following thermocycling conditions: an initial denaturation step at 95 °C for 5 min, followed by 45 cycles of 95 °C for 10 s and 60 °C for 30 s. U6 small nuclear RNA (U6 snRNA) served as the housekeeping gene for normalization. The relative expression levels of the target miRNAs were calculated using the 2^− ΔΔCt^ method [13].

#### Statistical analysis

GraphPad Prism version 9.00 for Windows (La Jolla, CA, USA) was used to create graphs and perform statistical analyses. The normality of quantitative data was assessed with the Kolmogorov–Smirnov test. For group comparisons, the Mann–Whitney *U* test was applied to non-parametric data, while the independent *t*-test was used for parametric data, depending on the distribution. Potential associations between continuous variables were analyzed using either Pearson’s or Spearman’s correlation tests, based on the data’s distribution characteristics. Results were reported as mean ± standard deviation (SD), with statistical significance defined as *P* < 0.05.

### Result

#### Characteristics of the study subjects

Table 1 shows the baseline data, demographics and laboratory measurements of the study participants. Osteoporosis and healthy control groups comprised of 9 (18%) males/41 (82%) females and 9 (18%) males/41 (82%) females, respectively. The mean age of the individuals in osteoporosis patients and healthy controls was 54.2 ± 7.5 and 53.7 ± 8.6, respectively. Hence, the groups were matched for sex and age.

The Z-score hip, T-score hip, Z-score spine, T-score spine, Z-score femur, T-score femur as well as spine BMD, hip BMD, and femur BMD of patients were significantly higher in patients than controls (Table 1).

#### Levels of Zn

**Fig. 1:**
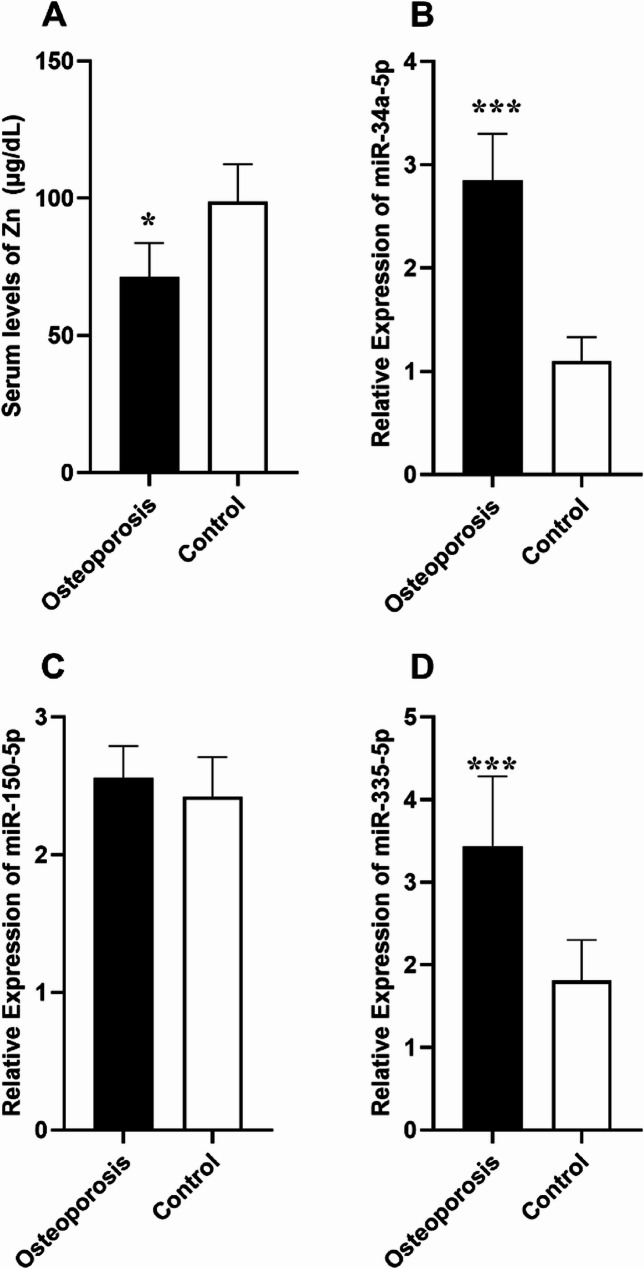
Bar graphs show serum levels of Zn (A) and serum transcript levels of miR-34a-5p, miR-150-5p, and miR-335-5p in Osteoporosis patients and healthy controls (*P < 0.05, ***P < 0.001)

Levels of Zn was significantly (*P* = 0.0047; Fig. 1.A) lower in the serum samples from osteoporosis patients (71.45 ± 12.24 µg/dL) in comparison to the healthy controls (98.79 ± 13.66 µg/dL).

#### Expression levels of miRNAs

Experiments revealed that the transcript level of miR-34a-5p (Fold change = 2.59, *P* = 0.008; Fig. 1.B) and miR-335-5p (Fold change = 1.90, *P* = 0.013; Fig. 1.D) was upregulated significantly in the serum samples of patients with osteoporosis compared to that of the healthy controls. However, no statistically significant differences were observed in serum transcript level of miR-150-5p (Fold change = 1.05, *P* = 0.509; Fig. 1.C) in the serum samples from osteoporosis patients compared to the healthy controls.

#### Association of Zn level and miRNA expression with patient’s data

The analysis (Table 2) revealed that transcript levels of miRNAs did not have statistically significant differences in osteoporosis patients according to the groups by gender (male vs. female), smoking status (smoker vs. non-smoker), residency status (urbane vs. rural), hypertension (yes vs. no), diabetes mellitus (yes vs. no), vitamin D supplement use (yes vs. no), calcium supplement use (yes vs. no), job (working/not working), marital status (married/single), education (educated/non-educated), familial history of Osteoporosis (Yes/No), bone fracture history (Yes/No), and familial History of bone fracture (Yes/No). However, analysis revealed reduced level of Zn in patients with bone fracture history compared to those without bone fracture history (68.91 ± 11.43 vs. 73.98 ± 12.51; *P* = 0.014). Nonetheless, when Zn level was compared between the other aforementioned groups, no statistically significant difference was detected.

**Table 2:** Comparison of MiRNA expression between patient groups

Patient data	miR-34a-5p	miR-150-5p	miR-335-5p
Sex (Male/Female)	1.25 (0.214)	1.03 (0.508)	1.20 (0.441)
Smoking (Yes/No)	1.08 (0.388)	0.93 (0.327)	1.17 (0.650)
Residency (Urban/Rural)	1.04 (0.207)	1.03 (0.771)	0.98 (0.659)
Hypertension (Yes/No)	1.11 (0.320)	1.09 (0.477)	1.06 (0.258)
Diabetes mellitus (Yes/No)	0.88 (0.493)	1.03 (0.710)	1.16 (0.610)
Vitamin D supplementation (Yes/No)	1.01 (0.366)	1.05 (0.905)	0.90 (0.707)
Calcium supplementation (Yes/No)	1.25 (0.928)	1.11 (0.564)	0.84 (0.359)
Job (working/not working)	1.02 (0.754)	1.00 (0.999)	0.97 (0.345)
Marital status (married/single)	0.99 (0.874)	1.11 (0.766)	1.05 (0.805)
Education (educated/non-educated)	1.07 (0.540)	1.02 (0.678)	1.03 (0.782)
Familial history of Osteoporosis (Yes/No)	1.12 (0.424)	1.20 (0.201)	1.14 (0.365)
Bone fracture history (Yes/No)	1.29 (0.122)	1.46 (0.184)	1.59 (0.071)
Familial History of bone fracture (Yes/No)	1.13 (0.401)	1.21 (0.321)	1.46 (0.122)

#### Correlation analysis

Analysis (Table 3) indicated that Zn levels in osteoporosis patients had significant negative correlation with transcript levels of miR-335-5p (*r*= −0.24, *P* = 0.037), but not miR-34a (*r*= −0.13, *P* = 0.253), and miR-150-5p (*r*= −0.03, *P* = 0.896).

**Table 3:** Correlation analysis between Zn and MiRNA transcription and the data of subjects with osteoporosis

Variable	Zn	miR-34a-5p	miR-150-5p	miR-335-59
	*r* (*P*)	*r* (*P*)	*r* (*P*)	*r* (*P*)
Age	0.16 (0.161)	0.15 (0.239)	0.07 (0.272)	0.11 (0.718)
BMI	0.11 (0.721)	0.13 (0.646)	0.14 (0.633)	0.07 (0.621)
Menarche age	0.08 (0.871)	0.13 (0.599)	0.14 (0.161)	0.1 (0.966)
Menopause duration	0.14 (0.404)	0.05 (0.343)	0.05 (0.876)	0.15 (0.819)
WBC count	0.20 (0.113)	0.15 (0.178)	0.12 (0.826)	0.11 (0.123)
Platelet count	0.11 (0.335)	0.06 (0.564)	0.14 (0.683)	0.11 (0.253)
Hemoglobin	0.12 (0.567)	0.07 (0.625)	0.12 (0.717)	0.07 (0.199)
RBC count	0.13 (0.775)	0.11 (0.161)	0.06 (0.209)	0.10 (0.559)
ALP	0.21 (0.071)	0.10 (0.649)	0.11 (0.451)	0.06 (0.622)
AST	0.15 (0.186)	0.06 (0.308)	0.07 (0.843)	0.06 (0.825)
ALT	0.12 (0.616)	0.14 (0.429)	0.07 (0.744)	0.06 (0.683)
LDH	0.13 (0.233)	0.10 (0.156)	0.14 (0.978)	0.11 (0.883)
CRP	0.19 (0.066)	0.14 (0.223)	0.12 (0.799)	0.12 (0.357)
ESR	0.16 (0.182)	0.07 (0.336)	0.12 (0.443)	0.11 (0.854)
FBS	0.13 (0.393)	0.08 (0.384)	0.10 (0.757)	0.13 (0.358)
TC	0.14 (0.459)	0.13 (0.804)	0.15 (0.411)	0.05 (0.949)
TG	0.05 (0.613)	0.14 (0.132)	0.06 (0.208)	0.09 (0.287)
LDL	0.08 (0.119)	0.11 (0.710)	0.05 (0.986)	0.06 (0.308)
HDL	0.08 (0.371)	0.12 (0.868)	0.14 (0.780)	0.05 (0.483)
Creatinine	0.08 (0.924)	0.15 (0.39)	0.06 (0.356)	0.09 (0.448)
BUN	0.11 (0.942)	0.07 (0.272)	0.08 (0.631)	0.08 (0.355)
Z-score hip	**0.28 (0.045)**	− 0.16 (0.743)	0.11 (0.903)	− 0.19 (0.269)
T-score hip	**0.30 (0.043)**	− 0.14 (0.272)	0.05 (0.684)	− 0.17 (0.196)
Z-score spine	**0.28 (0.020)**	− 0.19 (0.394)	0.07 (0.701)	− 0.13 (0.155)
T-score spine	**0.33 (0.009)**	− 0.21 (0.123)	− 0.15 (0.684)	− 0.16 (0.271)
Z-score femur	**0.31 (0.034)**	− 0.20 (0.082)	0.01 (0.148)	− 0.13 (0.375)
T-score femur	**0.35 (0.008)**	− 0.19 (0.123)	0.05 (0.718)	− 0.19 (0.166)
Spine BMD	**0.31 (0.017)**	− 0.18 (0.139)	− 0.15 (0.368)	− 0.15 (0.134)
Hip BMD	**0.34 (0.030)**	− 0.15 (0.327)	0.10 (0.601)	**− 0.19 (0.038)**
Femur BMD	**0.33 (0.021)**	**− 0.28 (0.044)**	0.11 (0.997)	− 0.10 (0.314)
Zn level	**-**	−0.13 (0.253)	−0.03 (0.896)	−0.24 (0.037)

*BMI*, Body-mass index, *WBC*, White blood cell, *ALP*, Alkaline phosphatase, *AST*, Aspartate aminotransferase, *ALT*, Alanine aminotransferase, *LDH,* Lactate dehydrogenase, *CRP*, C-reactive protein, *ESR*, Erythrocyte sedimentation rate, *FBS*, Fasting blood sugar, *TG*, Triglyceride, *LDL*, Low-density lipoprotein, *HDL*, High-density lipoprotein, *BUN*, Blood urea nitrogen, *BMD*, Bone mineral density. *Bold values show statistically significant comparisons

The correlation analysis was also performed between the Zn level as well as the transcript level of miRNAs and the clinicopathological characteristics of osteoporosis subjects (Table 3). It was found that Zn level had positive correlation with Z-score of hip (*r* = 0.28, *P* = 0.045), T-score of hip (*r* = 0.30, *P* = 0.043), Z-score of spine (*r* = 0.28, *P* = 0.020), T-score of spine (*r* = 0.33, *P* = 0.009), Z-score of femur (*r* = 0.31, *P* = 0.034), T-score of femur (*r* = 0.35, *P* = 0.008), spine BMD (*r* = 0.31, *P* = 0.017), hip BMD (*r* = 0.34, *P* = 0.030), and femur BDM (*r* = 0.33, *P* = 0.021).

In addition, a statistically negative significant correlation (*r*= −0.28 *P* = 0.044) was detected between transcript level of miR-34a-5p and femur BMD. There was also a significant negative correlation between transcript level of miR-335-5p and hip BMD (*r*= −0.19 *P* = 0.038).

#### ROC curve analysis

Receiver Operating Characteristic (ROC) curves were constructed to evaluate the ability to distinguish osteoporosis patients from healthy controls. The ROC curve visually represents the diagnostic performance of a binary classification system by plotting sensitivity (true positive rate) against 1-specificity (false positive rate) across varying threshold values. In this study, the Area Under the Curve (AUC) was calculated for miR-34a-5p, miR-150-5p, and miR-335-3p. The AUC is a critical metric for assessing the effectiveness of biomarkers in predicting outcomes such as osteoporosis. An AUC value of 1.0 indicates perfect diagnostic performance, while an AUC of 0.5 signifies no discriminative ability. Higher AUC values denote stronger predictive power of the miRNAs in distinguishing between osteoporosis and healthy states. The ROC curves for the three miRNAs were generated by systematically varying the threshold values defining positive and negative test outcomes, enabling a comprehensive analysis of their diagnostic utility.

**Fig. 2:**
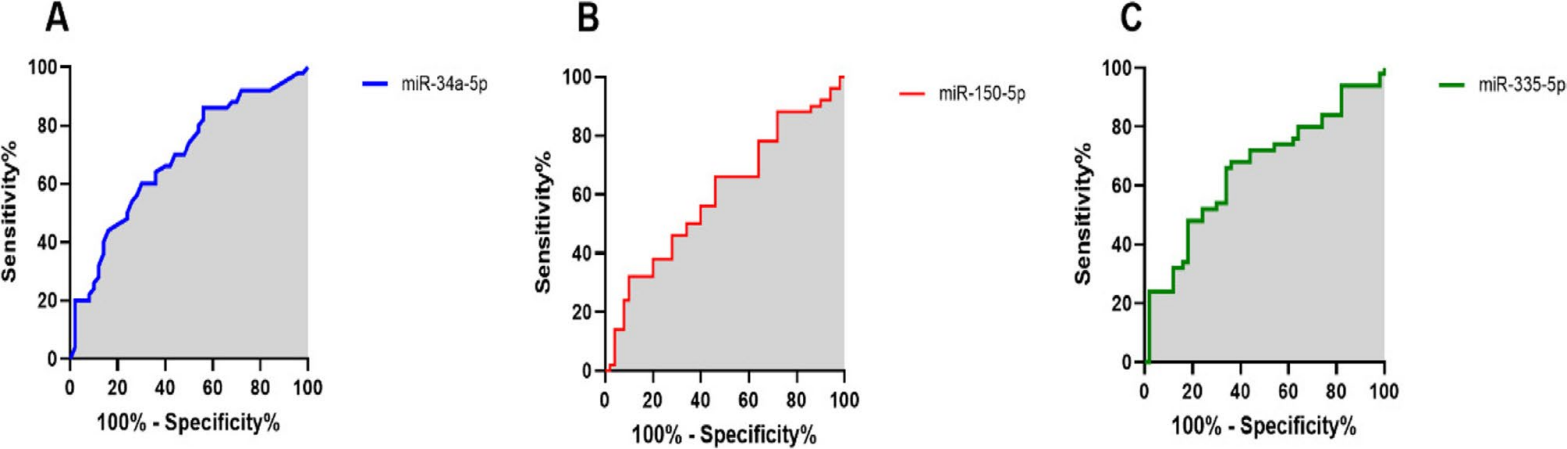
The ROC curves were designed and the AUCs were determined for miR-34a-5p (A), miR-150-5p (B), and miR-335-5p (C) to investigate the sensitivity and specificity of miRNAs in distinguishing osteoporosis and healthy controls (For miR-34a-5p; Sensitivity: 86, Specificity: 51, Optimal cutoff value: 1.119; For miR-150-5p; Sensitivity: 89, Specificity: 22, Optimal cutoff value: 3.19; For miR-335-5p; Sensitivity: 98, Specificity: 47, Optimal cutoff value: 2.739)

Based on our analysis, AUC for miR-34a-5p was 0.68 (95%CI: 0.58 to 0.79, *P* = 0.0012; Fig. 2.A), for miR-150-5p was 0.60 (95%CI: 0.48 to 0.71, *P* = 0.0848; Fig. 2.B), and for miR-335-5p was 0.73 (95%CI: 0.54 to 0.76, *P* = 0.0070; Fig. 2.C). Therefore, miR-34a-5p and miR-335-5p showed diagnostic accuracy in distinguishing between osteoporosis and healthy outcomes.

## Discussion

The primary aim of this study was to explore the potential relationship between Zn levels and the expression of miRNAs (miR-34a-5p, miR-150-5p, and miR-335-5p), focusing on their collective roles in bone metabolism and overall bone health among individuals with osteoporosis. In this study, Zn levels were significantly lower in the serum of patients with osteoporosis compared to healthy controls. This reduction in Zn was associated with upregulation of miR-335-5p, a miRNA known to influence pathways linked to osteoblast activity and bone turnover. Additionally, Zn levels exhibited a positive correlation with BMD, reinforcing its potential role in maintaining bone strength and structural stability.

Consistent with the majority of previous research, this study found significantly lower serum Zn levels in individuals with osteoporosis compared to healthy controls. In our findings, reduced Zn levels were correlated with poorer bone health in osteoporosis patients, as evidenced by lower BMD and other markers of bone strength. Furthermore, osteoporosis patients with a history of bone fracture had significantly lower levels of Zn compared to the patients without such history. This aligns with prior studies that have highlighted the importance of Zn in maintaining skeletal health. Moreover, clinical research has frequently shown positive associations between Zn biomarkers and bone health parameters, such as higher BMD and reduced fracture risk. The correlation between Zn deficiency and poor bone health in osteoporosis patients may stem from several mechanisms. Zn deficiency could impair the function of osteoblasts, which are crucial for bone formation, while promoting the activity of osteoclasts, which contribute to bone resorption. Additionally, Zn acts as a cofactor for various enzymes and signaling molecules that regulate bone metabolism, further emphasizing its multifaceted role in skeletal health. However, inconsistencies in the literature should also be acknowledged. While many studies report a positive relationship between Zn levels and bone health, others have found no significant association. These discrepancies may be attributed to differences in study populations, methodologies, and the biomarkers used to assess Zn status. Our findings underscore the potential of Zn as both a biomarker for assessing bone health and a therapeutic target for managing osteoporosis. Future direction should orient towards deciphering the precise molecular pathways linking Zn to bone metabolism, as well as investigate the efficacy of Zn supplementation as part of a broader strategy for osteoporosis prevention and treatment. Additionally, longitudinal studies are needed to establish causal relationships between Zn levels and bone health outcomes.

miRNAs have been implicated in bone remodeling. miR-34a-5p is associated with the regulation of osteogenic differentiation and bone remodeling [14]. Its expression might be elevated in osteoporosis patients due to its inhibitory effect on osteoblastogenesis and promotion of osteoclast activity, contributing to bone resorption [15]. Putative targets of miR-34a-5p include RUNX2, SATB2, and NOTCH1 [16], which are key regulators of osteoblast differentiation and function. miR-150-5p has also been implicated in osteoporosis and may be inversely correlated with bone health and BMD [17, 18]. It is suggested to target genes such as c-Myb and Akt1, thereby influencing osteoclastogenesis and immune cell-mediated bone remodeling [19]. miR-335-5p plays a role in bone development and homeostasis, with alterations in its levels reflecting disrupted signaling pathways in osteoporosis [20]. Some studies suggest increased levels of miR-335-5p due to its potential involvement in suppressing osteoblast differentiation, possibly through the downregulation of Dkk1 and RUNX2, both critical for Wnt signaling and osteogenesis [20]. In this study, we observed upregulation of miR-34a-5p and miR-335-5p in the serum of osteoporosis patients, suggesting these miRNAs may play a role in the pathophysiology of the disease. The increased expression of these miRNAs in osteoporosis patients may reflect their involvement in disrupting the balance between bone formation and resorption. The observed negative correlation between miR-34a-5p transcript levels and femur BMD highlights its potential role in femoral bone loss. miR-34a-5p has been implicated in inhibiting osteoblast differentiation and promoting osteoclast activity, which may lead to decreased bone formation and increased bone resorption. This aligns with previous studies suggesting that elevated miR-34a-5p levels contribute to bone fragility by shifting the remodeling process toward resorption. Similarly, the significant negative correlation between miR-335-5p transcript levels and hip BMD points to its detrimental effect on bone health in the hip region. miR-335-5p is known to modulate signaling pathways that impact osteoblast activity and matrix formation. Its upregulation may impair the ability of bone-forming cells to maintain or repair the structural integrity of the hip, a site commonly affected by osteoporotic fractures.

The analysis reveal that Zn level had negative correlation with miR-335-5p levels in the osteoporosis patients. Both Zn and miR-335-5p had also corrleation with BMD. It might be hypothesized that Zn and miR-335-5p had synergestic effect on bone health. Moreover, this might imply that Zn somehow might be involved in the regulation of miR-335-5p, thereby regulating the bone health in osteoporosis.

The analysis indicated a significant negative correlation between serum Zn levels and miR-335-5p expression in osteoporosis patients, suggesting a possible interplay between these two factors in bone health regulation. Additionally, both Zn and miR-335-5p were correlated with BMD, reinforcing their potential roles in maintaining skeletal integrity. This raises the hypothesis that Zn and miR-335-5p might exert a synergistic effect on bone health, potentially influencing bone remodeling processes together. This finding may also suggest a potential regulatory relationship in which Zn availability may influence the expression of miR-335-5p. Given the role of miRNAs in gene regulation, this dynamic might impact downstream molecular pathways crucial for bone metabolism. Zn is known for its role in bone health through its involvement in osteoblast activity, collagen synthesis, and mineralization processes. Meanwhile, miR-335-5p, based on existing evidence and our findings, may influence pathways that balance bone formation and resorption. The inverse relationship between Zn and miR-335-5p could indicate that low Zn levels trigger a compensatory upregulation of miR-335-5p, potentially as part of a stress response to impaired bone metabolism. However, this compensatory effect might also disrupt normal bone remodeling processes, contributing to the progression of osteoporosis.

A bulk of data testify that miRNAs may serve as biomarkers for diagnosis and prevention of osteoporosis [21]. The ROC curve analysis indicated that miR-34a-5p and miR-335-5p possess diagnostic potential for differentiating between osteoporosis patients and healthy controls. The AUC values for these miRNAs suggest their ability to discriminate with reasonable accuracy, highlighting their potential as biomarkers for identifying osteoporosis. MiR-34a-5p and miR-335-5p have been previously implicated in pathways associated with bone remodeling and metabolism. Their diagnostic accuracy may stem from their involvement in regulating gene networks critical for osteoblast and osteoclast activity, as well as their response to systemic factors affecting bone health. The observed upregulation of these miRNAs in osteoporosis patients aligns with their potential role in the pathophysiology of the disease, further supporting their utility as diagnostic tools. Additionally, the combination of these miRNAs with other clinical parameters, such as BMD, serum Zn level, or bone turnover markers, could enhance diagnostic precision. Integrating miR-34a-5p and miR-335-5p into a multi-marker panel may provide a more comprehensive assessment of osteoporosis risk and severity. It is also worth noting that the diagnostic value of these miRNAs might extend beyond distinguishing disease states; they could potentially serve as early indicators of bone deterioration before significant changes in BMD are detectable. This makes them valuable for screening at-risk populations, enabling timely intervention.

This study has several limitations that should be acknowledged. First, the relatively small sample size may limit the generalizability of our findings and the statistical power to detect more subtle associations. Larger, multi-center studies are necessary to validate the observed relationships and strengthen the conclusions. Second, the study design did not include mechanistic investigations and functional validation experiments to elucidate the underlying biological pathways linking Zn levels and miRNA expression with bone health. Understanding these mechanisms could provide deeper insights into the roles of these factors in osteoporosis. Third, the lack of follow-up data prevents us from evaluating longitudinal changes in Zn level, miRNA expression, and BMD over time or their predictive value for disease progression. Finally, we did not assess other potentially relevant confounding factors, such as dietary Zn intake, physical activity, or genetic predispositions, which may influence both Zn levels and bone health. Future studies addressing these limitations could provide a more comprehensive understanding of the interplay between Zn, miRNAs, and bone health in osteoporosis.

## Conclusion

In conclusion, this study highlights the significant associations between serum Zn level, miRNA expression (miR-34a-5p, miR-335-5p, and miR-150-5p), and BMD in patients with osteoporosis. We observed reduced Zn level and upregulated miR-34a-5p and miR-335-5p in osteoporosis patients compared to healthy controls, with strong correlations between these biomarkers and bone health parameters. Notably, the inverse relationship between Zn and miR-335-5p suggests a potential regulatory interaction, indicating their synergistic involvement in bone metabolism. Furthermore, ROC curve analysis demonstrated the diagnostic potential of miR-34a-5p and miR-335-5p, suggesting their utility as non-invasive biomarkers for distinguishing osteoporosis from healthy states. While these findings provide valuable insights into the molecular landscape of osteoporosis, further studies with larger sample sizes, longitudinal designs, and mechanistic investigations are needed to validate these results and uncover the pathways underpinning these associations. These efforts could pave the way for novel diagnostic and therapeutic approaches in osteoporosis management.

## Supplementary Information

Supplementary Material 1.

## Data Availability

The data that support the findings of this study are available from the authors but restrictions apply to the availability of these data, which were used under license from the Rafsanjan University of Medical Sciences for the current study, and so are not publicly available.
