# Supplementary material for: The role of serum zinc and MicroRNAs in osteoporosis: insights into bone metabolism and potential diagnostic markers
Source: BMC Musculoskelet Disord. 2025 Aug 9;26:772. doi: 10.1186/s12891-025-08970-9 (PMC12335165; doi:10.1186/s12891-025-08970-9)
Supplement: Supplementary file 1 — Supplementary Material 1. [file 12891_2025_8970_MOESM1_ESM.docx]

**Supplementary Table 1**. Primer pairs used to quantitative determination of miRNAs.

| **Primer** | **Primer sequence (5′→3′)** |
| --- | --- |
| miR-34a-5p - Forward | TACCACCGTCAGAATCG |
| miR-34a-5p - Reverse | GTCGTATCAGTGCAGGGTC |
| miR-150-5p - Forward | GTAGAGGGTTGGGAACATG |
| miR-150-5p - Reverse | GTCGTATCAGTGCAGGGTC |
| miR-335-5p - Forward | TTCTCGTTATTGCTTTTAG |
| miR-335-5p - Reverse | GTCGTATCAGTGCAGGGT |
| U6 - Forward | ACGTTGACAGCTATAATC |
| U6 - Reverse | GTCGTATCAGTGCAGGGTC |
